# Brazilian Clinical Psychologists’ Perceptions of Online Psychotherapy for Patients with Suicidal Behavior During the COVID-19 Pandemic: A Grounded Theory Study

**DOI:** 10.3390/ijerph22081284

**Published:** 2025-08-17

**Authors:** Natália Gallo Mendes Ferracioli, Elaine Campos Guijarro Rodrigues, Manoel Antônio dos Santos

**Affiliations:** Faculty of Philosophy, Sciences and Letters at Ribeirão Preto, University of São Paulo, Ribeirão Preto 14040-900, São Paulo, Brazil; elainecgr@hotmail.com (E.C.G.R.); masantos@ffclrp.usp.br (M.A.d.S.)

**Keywords:** suicidal behavior, suicide prevention, individual psychotherapy, COVID-19, telepsychology

## Abstract

Online psychotherapy for patients with suicidal behavior was considered inappropriate by the Brazilian Federal Psychology Council prior to the COVID-19 pandemic. Due to the need for physical distancing, this restriction was temporarily suspended. This study aims to analyze the perceptions of Brazilian clinical psychologists regarding online psychotherapy for patients with suicidal behavior in the context of the COVID-19 pandemic, to generate a theoretical understanding of this experience. It is a qualitative, exploratory, longitudinal study based on the Constructivist Grounded Theory framework. Ten clinical psychologists who conducted online psychotherapy for patients with suicidal behavior during the pandemic were interviewed at two moments, with an interval of approximately two years: the first round was conducted from December 2020 to March 2021, followed by a second round between November and December 2022. Data analysis led to four categories: (1) Reflecting on suicidal behavior; (2) Addressing specificities of online interventions; (3) Managing suicidal behavior in online psychotherapy; (4) Evaluating online psychotherapy for patients with suicidal behavior. The theoretical model “Pathways of care: main roads and access routes in online psychotherapy for suicidal behavior” was constructed. It was concluded that online psychotherapy for patients with suicidal behavior is complex and requires caution but is feasible and can be important in specific circumstances. This makes it an additional resource for suicide prevention and mental health promotion.

## 1. Introduction

The COVID-19 pandemic has been recognized as a factor that exacerbates vulnerability to suicidal behavior [1,2]. Suicidal behavior is a complex phenomenon influenced by both predisposing and precipitating factors, and the pandemic’s impact on this behavior is significant and should not be overlooked [2].

The extensive impacts of COVID-19 have led to the crisis being characterized as more than just a pandemic—it has been described as a syndemic [3]. Bispo Júnior and Santos [4] define a syndemic as the intrinsic interconnection of health issues, where the adverse social context exacerbates the effects, leading to more severe outcomes than would result from these phenomena occurring independently. The COVID-19 syndemic, therefore, involves a synergistic interaction among various chronic non-communicable diseases, infectious diseases, and mental health problems. Studies have reported an increase in symptoms of anxiety, depression, insomnia, and post-traumatic stress disorder during this period [5,6], all of which are associated with heightened risks of suicidal behavior [7].

Data on the prevalence of suicides or attempts during the pandemic are variable across different regions, countries, and population groups [8]. A study that compiled preliminary data from 21 countries [9] found no significant evidence of an increase or decrease in suicide rates at the beginning of the pandemic. Another study [5] associated suicides reported in global media during lockdowns with factors such as depression, loneliness, psychological stress, social media dependency, and COVID-19 infection. Islam [10] highlighted that in Bangladesh, contracting COVID-19 could lead to social stigma and elevate suicide risk. In Japan, a significant increase in suicides among women under 40 was reported [11]. A recent review focusing on Spanish-speaking countries found that suicidal ideation and suicide rates increased during the COVID-19 pandemic—and even in the post-pandemic period—particularly among children, adolescents, and young adult women from lower socioeconomic backgrounds. The most affected individuals often faced additional risk factors such as living in rural areas, experiencing mental health issues, unemployment, or the loss of family members to COVID-19 [12].

An international study identified a significant impact of the pandemic on youth suicide rates, with increases in depressive symptoms, anxiety, and suicidal behaviors, reporting alarming data from countries such as Italy (15% increase in suicide attempts among individuals under 19 in 2021), the U.S. (higher rates of ideation and attempts in 2020), Spain (7.4% rise in suicides in 2020 compared to 2019), Japan (49% increase during the second wave of the pandemic), and South Korea (over 35% of students experiencing emotional crises that could lead to suicide attempts) [13]. Macedo et al. [14] observed more suicides among the elderly during the pandemic, particularly those with pre-existing social vulnerabilities and emotional disorders. In addition, the purpose of a multicenter study is to investigate the association between suicidal behavior and COVID-19 itself, as the disease is multifaceted, with pathological features including chronic inflammation and neuropsychiatric symptoms [15]. Conversely, Cobo et al. [16] reported a decline in suicide rates during the pandemic. Panchal et al. [6] indicated that, following a brief decrease, suicide rates began to rise again from 2021 onwards.

Psychotherapy plays a crucial role in supporting individuals with suicidal ideation [2,17]. However, the COVID-19 pandemic has profoundly affected psychological practice by limiting physical proximity, necessitating a shift to online care [18,19,20]. Although the Guidelines for the Practice of Telepsychology [21] were established prior to the pandemic, the American Psychological Association (APA) rapidly adapted its position to accommodate the urgent need for remote clinical services and endorsed telepsychology even for practitioners without prior experience, stressing ethical flexibility, so psychologists were permitted to provide services via telehealth even without the necessary training in the crisis situation [22,23].

In Brazil, the practice of online psychotherapy has been historically regulated by the Federal Council of Psychology (CFP). Beginning in the early 2000s, online psychological services in Brazil were regulated in an experimental and highly restricted manner. Resolution No. 3 of the Federal Council of Psychology [24] permitted Information and Communication Technology (ICT)-mediated care only within research contexts. Subsequent regulations [25,26] allowed limited online psychological guidance—distinct from psychotherapy—and imposed strict conditions, such as a maximum of 20 sessions and use of certified platforms based in Brazil.

A substantial shift occurred with Resolution No. 11 of the CFP [27], which marked a paradigm change by granting full professional autonomy regarding the adequacy of methods and techniques used in ICT-mediated psychological services. This resolution officially authorized online consultations and treatment. However, it still considered online care for emergencies and urgent cases, including individuals with suicidal behavior, as inadequate.

To legally provide such services, psychologists were required to register in the national e-Psi system by signing a formal declaration. While this is not a special license, it represents a regulatory obligation. The COVID-19 pandemic prompted Resolution No. 4 of the CFP [28], which temporarily suspended the restrictions on online care for emergency situations, also allowing professionals to provide remote support without waiting for formal approval.

This regulatory flexibility led to a sharp increase in professional registrations: over 39,000 new e-Psi registrations were recorded in just one month at the beginning of the pandemic—surpassing the total from the previous 15 months [29]. These numbers reflect the magnitude of change experienced by psychologists in adapting their practices to the pandemic context. Many professionals transitioned to online modality without any prior experience, which required a rapid reinvention of technical aspects of psychotherapy. ICT mediation demanded the swift development of new therapeutic strategies, often guided solely by the therapist’s internal clinical framework, to establish new forms of connection under radically altered conditions of encounter [30].

Importantly, these professionals were not only adjusting their clinical methods but also personally affected by the pandemic’s emotional, social, and familial impacts. Psychologists reported the emotional strain of supporting patients in distress while themselves experiencing fear, uncertainty, and helplessness amid a broader sociopolitical and public health crisis [30,31,32]. The shared experience of trauma challenged the boundaries between therapist and patient and reshaped the meaning of psychological care in times of collective emergency.

This research was driven by the ethical and professional imperative to provide empirical and theoretical contributions that may support the development of guiding references for psychotherapists engaged in suicide prevention, intervention, and postvention during the unprecedented global health crisis triggered by the COVID-19 pandemic. The broader purpose of this study is to reflect on clinical psychologists’ roles in assessing and managing suicide risk in online settings during the period of self-isolation, based on their lived professional experiences.

Such knowledge is essential, as it is considered crucial for professionals to be both theoretically and technically equipped to assess suicide risk and appropriately manage psychological crises and emergencies. This includes mobilizing community resources to implement adequate measures in high-risk or extreme vulnerability scenarios. Banerjee et al. [1] state that the availability of online interventions served as a preventive measure against suicidal behavior during the pandemic.

Jones, Byrne, and Carr [33] argue that the context brought about by the COVID-19 pandemic offers an opportunity for researchers to critically reflect on the directions they truly wish to pursue—emphasizing meaningful actions over mere discourse. In this regard, the present study is concerned with producing knowledge that contributes to psychotherapeutic clinical practice, which is undergoing rapid transformation.

Given these premises, and considering that professionals were required to offer a type of care not previously anticipated for this form of psychological vulnerability, this study aims to analyze the perceptions of Brazilian clinical psychologists regarding online psychotherapy for patients with suicidal behavior in the context of the COVID-19 pandemic, to generate a theoretical understanding of this experience.

## 2. Methods

### 2.1. Study Design

A qualitative, exploratory, and longitudinal study was conducted, adopting the methodological framework of Constructivist Grounded Theory (GT). This approach views theories as constructions of reality that illuminate complex issues and expand interpretative repertoires on phenomena. It is particularly useful for research in areas where existing theory is still in its early stages, insufficient, or even absent [34]. Such is the case with the focus of this study, given that prior to the pandemic, online psychotherapy for individuals with suicidal behavior was considered inappropriate in Brazil and, therefore, should not have been conducted. This restriction was suspended during the pandemic, creating a pressing need for studies to support and guide professional practice under these new conditions, as previously explained.

### 2.2. Participants

The study involved ten Brazilian clinical psychologists. Inclusion criteria required participants to be practicing psychologists in the State of São Paulo, Brazil, who provided online psychotherapy to patients with suicidal ideation, plans, and/or attempts during the pandemic. This region was selected because it has the largest number of active psychologists in the country. In Brazil, the professional practice of psychologists is regulated by the Federal Psychology Council (CFP), a public law autarchy with administrative and financial autonomy, responsible for overseeing, guiding, and supervising professional practice. To accomplish this, the CFP is subdivided into various regional units, referred to as Regional Psychology Councils (CRP). Of the 24 Regional Psychology Council (CRP) sub-offices in Brazil, it is noteworthy that CRP-06 (São Paulo) accounts for over 30% of all registered psychologists in the country.

Exclusion criteria included psychologists who were not registered with CRP-06, those without an active CRP, and clinical psychologists who exclusively provided face-to-face psychotherapy for patients with suicidal behavior during the pandemic.

The sample consisted of seven women and three men, aged 29 to 69, with clinical experience ranging from four to 30 years at the start of the study. In terms of racial/ethnic identity, nine participants identified as White, and one as Brown. All participants were native Brazilians. Socioeconomically, participants ranged from middle to upper class. They graduated from public (*n* = 2), private (*n* = 6), or confessional (*n* = 2) institutions. Their further training included specializations (*n* = 6) and stricto sensu postgraduate courses (*n* = 4). Psychoanalytic and phenomenological-existential theoretical approaches predominated among them. Six participants had no prior experience with online therapy before the pandemic, while four had occasionally engaged in online sessions under exceptional circumstances with patients typically seen in person.

### 2.3. Instruments

The data collection was divided into two phases. In the first phase, the following instruments were employed: (1) a sociodemographic information form; (2) a thematic semi-structured interview guide, initiated with the question: “I would like you to describe your experience in providing online care to patients with suicidal behavior during the COVID-19 pandemic”; and (3) a field diary. Follow-up questions addressed topics such as prior experience with suicidal patients, the transition to online care, challenges and facilitators, adjustments, strategies employed, and future outlooks.

In the second phase, data collection included a field diary and a second interview guide. The central question of this phase focused on participants’ views on online psychotherapy at a time when it was no longer the only option. Specific topics included perceptions and evaluations of online care for individuals with suicidal behavior, intentions to continue using this format in the future, and the reasons for such decisions.

### 2.4. Procedure

Constructivist Grounded Theory recommends simultaneous data collection and analysis, allowing for the continuous adaptation of the research process based on emerging insights [34]. Initial sampling aimed to generate rich data for subsequent theoretical sampling, which then focused on developing categories linked to theory construction. In GT, theoretical sufficiency is reached when no new properties or theoretical insights emerge, rather than through mere data repetition. In this study, theoretical sufficiency was achieved after two rounds of interviews with the ten participants.

Participants were initially contacted via WhatsApp^®^ (Menlo Park, CA, USA; iOS version 2.20.130 in 2020/2021 and 22.24.81 in 2022) receiving an introduction to the researcher, the source of the contact, and the study’s objectives. The same participants comprised the theoretical sample in the second round. To preserve anonymity, participants selected fictitious names.

In the first round, 10 individual interviews were conducted via video call, with consented recordings taking place between December 2020 and March 2021. Following each interview, non-verbal cues, emotional expressions, and the researcher’s reflections were documented in a field diary. Interviews lasted between 40 and 80 min, with an average duration of 62.7 min, resulting in 627 min of recorded material. The researcher fully transcribed the interviews, producing 193 text pages that formed the initial corpus of the study.

The second round followed the same procedures, conducted later in the pandemic, between November and December 2022. These interviews were shorter, lasting between 8 and 28 min (average 16.5 min), totaling 165 min. The transcripts from this round added 50 pages to the dataset.

All 20 interviews were analyzed, totaling 792 min of recording and 243 pages of transcription, and was conducted according to the methodological steps of Constructivist Grounded Theory [34]: (1) open coding, line by line, in which codes were assigned to the data, which culminated in the construction of 493 initial codes; (2) focused and selective coding, in which the initial codes were used to classify and synthesize information, leading to the construction of four categories and eight subcategories. A total of 35 memos documented ideas, interpretations, and theoretical insights during this process. The analyses were discussed among the three evaluators. Constant comparison of analytical elements—including interviews, diaries, memos, codes, categories, and subcategories—was employed to identify patterns and make the necessary refinements. Atlas.ti (Berlin, Germany; version 22.0.11.0) software facilitated the coding process and data management, which enhanced methodological rigor and enabled multiple forms of analysis. The researcher utilized the software to import the interview data, which offered various tools for organizing and editing the material. Through in-depth readings, codes were systematically developed and structured, later exporting the selected excerpts into spreadsheets that supported the construction of categories and subcategories.

Data analysis was discussed among the three authors. The first author conducted the interviews, transcriptions, and analysis, while codes, categories, and subcategories were discussed with the co-authors for input and with members of the research lab with expertise in qualitative research. Additionally, four study participants validated the theoretical model constructed from the data analysis.

### 2.5. Ethical Considerations

This study was approved by the Research Ethics Committee of the Faculty of Philosophy, Sciences and Letters at Ribeirão Preto, University of São Paulo (approval number: 4.476.733, CAAE: 40420620.5.0000.5407) and adhered to the ethical guidelines set forth in Resolution No. 466/12 on research involving human subjects [35]. Participants received an Informed Consent Form (ICF) via email, which they printed, signed, and returned in digital format. This procedure aligns with the Guidelines on Ethics in Research in Virtual Environments [36].

### 2.6. Positionality Statements and Reflexivity

In constructivist GT, researchers must engage in reflexivity, acknowledging that their analyses are shaped by personal values and that data are co-constructed through interactions with participants [34]. Thus, the theory generated reflects the researcher’s interpretations rather than an independent reality, necessitating consideration of the evaluative dimension.

The first author, a researcher and clinical psychologist, has extensive experience treating individuals with suicidal behavior in hospital and clinical settings. She began practicing online psychotherapy in 2018, but it was only during the pandemic that she treated patients with suicidal behavior remotely. In the first year of the pandemic, she conducted all sessions online, including with a patient exhibiting suicidal ideation, as permitted by temporary changes in the CFP Resolution [28]. This experience motivated her interest in the research. The second author is a researcher and clinical psychologist who also provided online psychotherapy during the pandemic, and the third author is a psychologist and researcher who has substantial and extensive academic experience.

Some participants were previously known to the first author, as part of her professional network, while others were not. The study adheres to the COREQ (Consolidated Criteria for Reporting Qualitative Research) guidelines [37].

## 3. Results

As previously stated, the initial line-by-line coding of the transcripts generated 493 codes, which led to the identification of eight subcategories in the focused coding phase. These subcategories were subsequently grouped into four overarching categories: (1) Reflecting on Suicidal Behavior; (2) Addressing Specificities of Online Interventions; (3) Managing Suicidal Behavior in Online Psychotherapy; and (4) Evaluating Online Psychotherapy for Patients with Suicidal Behavior. A detailed analysis of these categories culminated in the construction of the theoretical model titled “Pathways of Care: Main Roads and Access Routes in Online Psychotherapy for Suicidal Behavior” (Table 1).

### 3.1. Reflecting on Suicidal Behavior

#### 3.1.1. General Experiences in Managing Suicidal Behavior

Nine participants highlighted a lack of specific training during their undergraduate or postgraduate studies, necessitating self-directed learning and supplementary training. Débora noted: “Nobody addresses suicide in undergraduate courses, to the extent that it’s one of the criteria in the university clinic: if there’s a suicide-related demand, we cannot provide care”.

Regardless of the pandemic, the importance of the therapist–patient bond in the psychotherapeutic treatment of individuals with suicidal behavior was emphasized by the professionals. They also discussed their theoretical and technical knowledge, covering aspects such as risk and protective factors, assessment strategies, therapeutic interventions, and ethical considerations. Differences in management strategies between hospital/emergency contexts and private practice settings were also highlighted.

The clinical psychologists also explored the impact of bereavement and the consequences of suicide on survivors, including the professionals involved in patient care. Two participants shared experiences of losing patients to suicide, reflecting on the profound emotional impact. Eduardo stated: “[…] he died by suicide and remained in my thoughts for three or four years. It was deeply painful, causing grief and professional pain, which I had to process”.

#### 3.1.2. Suicidal Behavior During the Pandemic

Participants underscored the critical need to recognize suicide risk in patients during online sessions amid the pandemic, even though this task might appear straightforward. The professionals stressed the importance of vigilance for subtle indicators of suicide risk, which allows for timely intervention and appropriate management.

Professionals identified passive exposure to COVID-19 as a potential indicator of a desire to die, whether conscious or subconscious. Manoel shared: “It has happened that a patient clearly expresses: ‘If I die, then I’m dead; it’s better to be dead than to endure this situation’ […] Death is so threatening that, to escape it, one might prefer to die completely”. He also gave another example: “A patient might spend the week obsessively avoiding contamination but then engage in risky behavior over the weekend, which, to me, signals a suicidal tendency”.

This scenario differs from managing explicit suicidal behavior, such as urgent/emergency situations in which the patient articulates suicidal ideation, describes plans during the session, seeks immediate help, or reports a recent suicide attempt. Débora shared: “The patient informed me via text, sending a message about the attempt she had made. Fortunately, she aborted the attempt midway and reached out to me”.

The incidence of suicidal ideation or attempts during the pandemic varied, according to the professionals’ experiences: some reported an increase, others a decrease, while some observed no change. Even those working in hospitals, emergency services, and private practices reported differing perceptions of the number of patients presenting with suicidal behavior.

### 3.2. Addressing Specificities of Online Interventions

#### 3.2.1. Challenges, Difficulties, and Insecurities

Initially, some of the interviewed professionals were reluctant to adopt online modalities for managing suicidal behavior. However, as the pandemic persisted, they recognized the necessity of offering this modality to patients. Numerous insecurities were reported: concerns about the effectiveness of interventions, the maintenance and quality of the therapeutic bond, changes in the therapeutic setting, and issues related to privacy, confidentiality, and ethical considerations. Additionally, participants expressed feelings of reduced control, increased powerlessness, diminished non-verbal communication, and session interruptions. Participants reported: “My biggest concern, which left me with doubts, was about the bond. Are the effects of the work the same?” (Débora); “Sometimes the [internet] connection was lost, and these interruptions made it difficult for the person to resume their train of thought. Children interrupted sessions, and there were challenging moments” (Sophia).

Challenges included limited access to healthcare systems in patients’ locations and inadequate psychosocial support: “The patient’s sister was the only person in town who was part of her support network. They had conflicts, but she was our only alternative at the time” (Julia); “When I finally mapped out the nearest mental health service, they were no longer accepting patients. I tried calling multiple times, emailing […]. Our experience across Brazil has shown us how lacking these resources are” (Thais); “In my city, it happened often: the patient would arrive at the hospital, and they would rush to send them away. Sometimes, I was very worried” (Sophia).

Managing the risk and threat of suicide during online psychotherapy sessions presented significant challenges, generating anxiety and fear. The physical distance compelled professionals to act more promptly and decisively than they might have in in-person sessions, leading to doubts about the appropriateness of their actions. Julia illustrates this vividly:

There was a situation where the patient, during our session, said: ‘I’m going to jump off the balcony.’ And then I thought: ‘Oh my, what do I do now?’ She lives on the sixth floor! Maybe that wouldn’t have happened in my office, which is a safe space. Being far away is much more challenging; we tend to feel we have more control when we’re there with the patient.

#### 3.2.2. Psychologists’ Feelings on the Online Management of Suicidal Behavior

The psychological repercussions for psychotherapists managing individuals with suicidal behavior via online sessions included feelings of discomfort, fear, distress, anguish, worry, tension, insecurity, overload, and despair. Julia described her experience: “At times, online, I felt apprehensive. What tension, what despair,” while Thais expressed her distress when dealing with patients who were not receiving adequate care: “The family member manages to take the patient to the mental health service, and they tell them not to come back until Monday. And then it was Thursday, Friday, Saturday, Sunday in gigantic distress. Gigantic”.

In some cases, clinical psychologists felt more powerless when managing suicidal behavior online compared to in-person interventions. They frequently expressed a desire to be physically present with the patient during critical situations. Sandra shared her concerns:

I think online makes you a bit more powerless, it’s as if you can’t even offer physical containment, if necessary. You feel incapable in that sense. It’s distressing: ‘What if he hangs up and tries again?’ In the office, you’d be more aware of how he is physically. The assessment could be richer. I remember the feeling online with this patient. Terrifying. I confess I don’t even like remembering that patient, you know?

Four participants experienced suicide attempts by patients during the pandemic, which generated guilt and sorrow. However, they also expressed a sense of hope and relief upon realizing that online management was feasible.

### 3.3. Managing Suicidal Behavior in Online Psychotherapy

#### Strategies and Resources Used

Participants in this research utilized established theories and techniques, adapting them to effectively address suicidal behavior through ICT. Psychologists also emphasized the necessity of training to effectively provide crisis interventions.

Successful strategies included ensuring the patient about the professional’s availability for emergencies, offering immediate care during crises, conducting additional sessions, and using text messages for support until the scheduled appointment. In certain situations, professionals proposed alternative times of the day (for example, early in the morning or late at night) to maintain confidentiality and protect from potential exposure due to the presence of other people in the patients’ homes. In more extreme cases, they extended session durations and canceled subsequent appointments. Eduardo stated:

I see a lot of patients with suicidal ideation and attempts, and the online modality for these patients is notably interesting. It emphasizes accessibility, making the patients feel closer to the therapist, which is fundamental. It’s like saying, “If I need you, I can call, and you’ll find an appointment for me quickly, or you’ll provide some reassurance word to help me a bit”.

During online sessions, the importance of attentively listening to the patient’s suicidal ideation, validating their suffering, addressing the ambivalence (when present) between the desire for life and the desire for death, demonstrating concern for the patient’s wellbeing, and providing reassurance was emphasized. Psychologists frequently intervened verbally, adopting a more concrete or directive approach: “In these situations, I need to work in a more concrete manner. It’s more about managing the moment than reflecting on it. Reflection occurs after I have addressed the emergency” (Julia).

The necessity of advising patients to utilize their psychosocial support networks and the ethical implications of breaking confidentiality in suicide risk scenarios were highlighted: “I requested someone present during at least one online session” (Thais). Additionally, they emphasized the need to map local public health and safety resources (such as emergency care centers, psychiatric support centers, psychiatric hospitals, clinical psychiatrists, police, and fire departments) to facilitate potential in-person interventions: “I made sure to clarify to the patient: ‘If you need anything, reach out to your support network’” (Thais). The interviewed psychologists made referrals to clinical psychiatrists when necessary.

There were instances when psychologists needed to inquire about the patient’s location during crises and assess access to lethal means (high-rise apartments, objects, medications, chemicals, etc.). In extreme circumstances, some professionals permitted face-to-face meetings, overriding physical distancing guidelines, while others continued with online consultations even after suicide attempts.

Below is a detailed account of how psychologist Julia managed a suicidal crisis via video call:

There was a situation when the patient was in significant conflict. That day, she requested an earlier session. During our session, while I was on the phone, she went to look out on the balcony. I could see that she was very distressed and needed support. I started to cancel my appointments with other patients. I asked for her sister’s contact information, as she was her only support person […] Everything I asked her to do, she did: “Sit in the chair and look at me.” I tried to foster a sense of closeness: “We’re here together,” in an effort to ground her. Despite feeling insecure, I endeavored not to convey that to her, as in that moment she needed reassurance. She expressed despair, saying: “I’m done. I’m tired of struggling without finding a way out. It’s easy; I just need to jump, and I won’t feel a thing.” I replied: “But you’re already feeling too much. You’re trying to escape from something we cannot avoid. Let’s just sit here together and acknowledge that we’re in this together; you’re not alone.” However, she wouldn’t engage. She would pace by the window and return, saying: “Julia, I can’t, I can’t, I can’t.” I asked: “Can’t what?” I was trying to discern if she was trying to jump, because I couldn’t see her at those moments. I asserted: “Let’s think this through; I can see you’re very agitated, and you might do something you genuinely don’t wish to do. I need your address.” I knew she lived nearby, and I began to contemplate what I would do if I couldn’t reach her sister: I would call the police or respond in person with their assistance. I opened WhatsApp on my computer to communicate with her sister, telling her about the situation. Imagine me attending to the patient while managing communication with her sister on separate devices. Such tension! I felt as if I were juggling multiple roles simultaneously. Her sister arrived after roughly 45 min, and I then spent another hour with both of them.

After resuming in-person sessions, many psychologists continued to utilize online resources, such as text messages and immediate sessions during severe crises.

### 3.4. Evaluating Online Psychotherapy for Patients with Suicidal Behavior

#### 3.4.1. Perceived Facilities and Advantages

As previously discussed, study participants noted that online therapy enhances access to support during crises—an advantage considered crucial, as it enables rapid intervention without requiring physical displacement. Clinical psychologists also recognized that this modality may strengthen the bond between therapist and patient, enriching their emotional connection:

In a sense, it brings them closer. It instills confidence in knowing that if they need assistance in an emergency, there is someone available. On occasions when a patient was in crisis and planning an attempt, she would text me, and I would promptly respond (Paula).

Participants also highlighted the advantage of receiving more immediate online supervision regarding crisis management: “Knowing the supervisor was readily available to respond to messages and that I wasn’t alone, having that support helped me to think through the best course of action” (Thais).

Participants identified several advantages as exclusive to online psychotherapy—benefits not typically present in in-person contexts. These persisted even after the end of the health emergency triggered by the COVID-19 pandemic.

For introverted or reserved individuals, online therapy facilitated help-seeking behavior. The physical distance reduced feelings of personal exposure and fostered a sense of safety, helping to alleviate the taboos and stigma associated with social perceptions of individuals experiencing suicidal behavior: “If you think that the person is there for something therapeutic, to talk a little, to have something that is very comfortable for them, I imagine that online is much better for them to protect themselves” (Milena); “I firmly believe that online therapy enhances access for individuals in need. I have no doubt about it” (Manoel).

Moreover, participants noted that the online format supported the continuity of psychotherapy. They identified critical moments in the therapeutic process when psychological defenses were heightened, leading patients to prefer online sessions even when in-person attendance was possible. This was particularly relevant for patients with severe depression who struggled with basic daily activities yet could still engage in therapy remotely until they felt prepared for face-to-face meetings:

A patient requested to see me exclusively online, even though she could physically come to my office. She was in a severely depressed state, unable to rise from bed. I provided online consultations for her because she truly couldn’t leave her house (Julia).

According to the professionals interviewed, online psychotherapy offers additional advantages for patients exhibiting suicidal behavior: it enables individuals residing in regions with limited access to specialized mental health services—or in smaller towns where familiarity with local psychotherapists is common—to receive quality treatment that would otherwise be inaccessible.

#### 3.4.2. Disadvantages Identified

Some clinical psychologists noted a sense of something lacking in online modality, perceiving it as more limited and creating symbolic barriers to reaching patients. It was frequently reported that ICT mediation can lead to increased fatigue and pose additional challenges in urgent situations, leading to a stronger desire for in-person contact with the patient. Milena articulated:

It feels much more ethereal online. It’s dispersed; and I find it very challenging to connect with the other person […] It’s not impossible […] yet something remains elusive. While this also occurs in person, the virtual arena amplifies what you can’t touch in the other person. It’s as if something becomes very open.

#### 3.4.3. General Assessment

Reflecting on their experiences during the COVID-19 pandemic, participants of this study perceived online psychotherapy for patients with suicidal behavior to be more complex and demanding of caution; nevertheless, it is deemed therapeutic and viable: “It was possible, but very challenging” (Julia). A comparison was drawn between the online support provided to individuals with suicidal behavior and the operations of the Centro de Valorização da Vida (CVV), where trained volunteers offer emotional support to those in urgent psychological distress, many of whom are at risk of suicide:

In the 1980s, I volunteered with CVV. We communicated over landlines, never seeing the person. The advent of online support reminded me significantly of that experience. While it was a different role than a psychologist’s, people helped each other and connected over the phone (Hélio).

Participants in this study observed that the skill and confidence exhibited by psychologists in employing ICT for online sessions influence patient engagement. The interviewed psychologists also concurred that the quality of the therapeutic bond between the psychologist and the patient is critical for the success of online consultations; however, they maintained that online sessions did not undermine the establishment and maintenance of this bond, attributing drops in attendance to other factors:

It’s likely that these patients wouldn’t have continued even in-person scenarios. When individuals are grappling with pain they cannot endure, pursuing therapeutic engagement involves touching wounds that inherently provoke discomfort. If patients successfully navigate through their most painful experiences and develop a manic defense, the tendency is to withdraw (Manoel).

Psychologists who participated in this study have asserted that ICT mediation limits the opportunity for physical presence, particularly for socially isolated patients, believing that in-person contact can provide greater comfort for individuals with suicidal behavior than virtual interactions. However, they also recognized that “entering” a patient’s home through online sessions might foster closeness and intimacy. Regarding accessibility between patient and therapist, professionals noted potential distortions, including boundaries in communication outside of scheduled sessions.

All clinical psychologists interviewed in this study considered online care for individuals with suicidal behavior as an essential resource during the pandemic. However, they acknowledged that quality care requires professionals to rely on support networks, including personal psychotherapy (often conducted online), peer supervision, and ongoing education.

During the second round of interviews, professionals provided retrospective evaluations of online psychotherapy for patients with suicidal behavior throughout the pandemic. They reiterated that despite some challenges, their patients adapted and improved: “I believe the online modality, particularly for this population, functions effectively” (Eduardo); “It operates equivalently” (Débora); “It might be slightly uncomfortable at times, as you miss out on observing the person’s behavior and body language, but I believe it’s manageable” (Paula).

All psychologists articulated their intention to continue offering online care beyond the pandemic. Nevertheless, some professionals conveyed a preference for in-person interactions with individuals experiencing suicidal behavior, reserving online formats for specific scenarios or when no other options were available. Other participants opted to defer to patient preferences, taking individual circumstances into account: “I personally prefer addressing suicide-related requests in person. However, my perspective has evolved; if the individual is open to engaging online, that’s a viable pathway to initiate therapy” (Débora).

### 3.5. Pathways of Care: Main Roads and Access Routes in Online Psychotherapy for Suicidal Behavior

The experiences of Brazilian clinical psychologists during the COVID-19 pandemic culminated in a theoretical model for online psychotherapy targeting patients with suicidal behavior, with applicability extending beyond the pandemic context. Participants thoroughly evaluated and explored the implications of this approach while projecting its future use. Despite numerous discussions and theoretical reflections on the importance of providing ICT-mediated mental health services during the pandemic, to date no empirical studies have been found that address the implementation and effects of this practice. This is the original contribution of this research, which sought to produce knowledge capable of enriching the development of online clinical practice, which is undergoing a remarkable transformation.

**Figure 1:**
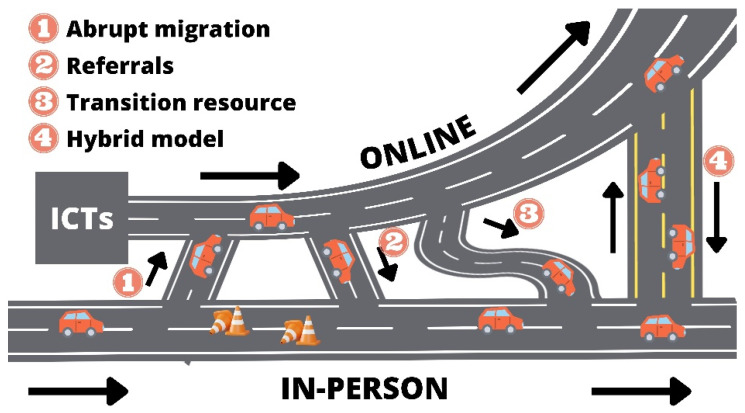
Representation of the theoretical model “Pathways of Care: Main Roads and Access Routes in Online Psychotherapy for Suicidal Behavior”.

To enhance understanding of the proposed theoretical model, a visual analogy was created, representing online psychotherapy and traditional in-person therapy as adjacent roads interconnected by various access routes (Figure 1). It is important to clarify that the four access points depicted in the figure do not correspond directly to the previously presented categories. Rather, they illustrate the theoretical model developed through the integration of those categories, which will be appropriately explained and detailed in the following section.

Initially, the pathway to in-person consultations was well-established and familiar. Over time, online psychotherapy emerged as a narrower alternative, deemed unsuitable for managing cases involving suicidal behavior. The COVID-19 pandemic introduced challenges (illustrated as traffic cones), prompting professionals to transition to online modalities (Access 1 in Figure 1), which became the primary route during that period, including cases involving suicide risk.

Despite the rapid migration to online psychotherapy for individuals exhibiting suicidal behavior, concerns arose regarding its efficacy. Some patients might not benefit from this approach and would be better served through in-person referrals. In the post-pandemic phase, practitioners will continue to provide online care for patients with suicidal behavior but will refer them to in-person psychotherapy if they identify any inadequacies. In the theoretical model, these referrals for face-to-face care are depicted as a direct connection route (Access 2 in Figure 1).

Upon examining both primary pathways (in-person and online psychotherapy) and their interconnections, clinical psychologists identified an opportunity for support through ICT mediation as an entry point to in-person psychotherapy (Access 3 in Figure 1). This approach is considered beneficial for patients facing temporary barriers, including physical health issues, limited mobility, psychological resistance, or serious psychiatric conditions such as phobias and severe depression, which preclude attendance at in-office therapy sessions.

As health restrictions were suspended, allowing for the resumption of in-person sessions, some patient–therapist pairs opted for this format while others continued with online sessions, with the option to seek emergency services when necessary. The decision regarding the preferred pathway was a collaborative process between the professional and the patient, weighing the advantages and disadvantages of each modality for individual cases.

A situation commonly reported in the experiences of study participants was the implementation of hybrid psychotherapy, which combined in-person and online sessions for the same patient to enhance adherence and continuity in therapy. In the theoretical model, this scenario is represented by a two-way street that connects the two main roads (Access 4 in Figure 1), facilitating traffic in both directions.

Moreover, some participants noted receiving patients for online psychotherapy who were uncomfortable disclosing suicidal ideation to therapists in their local area due to concerns about moral judgments and potential breaches of confidentiality. Additionally, in regions lacking specialized professionals, online psychotherapy became the sole option for treating patients with suicidal behaviors and was therefore critically important. In these contexts, online psychotherapy has evolved from a narrow pathway into a broad main road.

The illustration of the theoretical model also represents an upward curve, symbolizing the growth anticipated by the professionals interviewed, consistent with the existing literature. Regardless of the pathways taken, both routes ultimately aim toward the same goal: care, promotion of mental health, and the prevention of suicidal behavior.

## 4. Discussion

This study investigated how Brazilian clinical psychologists addressed suicidal behavior in online psychotherapy during the COVID-19 pandemic. The participants’ narratives suggest a complex process marked by challenges, adaptations, and critical reflections. Many of their experiences align with international findings and underscore the relevance of examining how mental health professionals adjusted their practices to deliver care in the face of an unprecedented global crisis.

International research indicates that psychotherapists experienced profound disruptions in both professional practice and personal well-being during the COVID-19 pandemic. Key challenges included the abrupt shift from in-person to online sessions, increased workloads, and a surge in patients’ psychological distress. These pressures often resulted in emotional exhaustion and a sense of weakened therapeutic connection [38]. The physical distance inherent in online psychotherapy intensified professionals’ sense of vulnerability during crises. This perception contributed to distress and fear, especially when rapid decision-making was required without being physically present. The literature confirms that online contexts can generate similar reactions among professionals, particularly when dealing with high-risk patients [19,39,40,41]. Nevertheless, many therapists also reported gains in flexibility, digital skills, and a growing appreciation for remote modalities—revealing a paradoxical dynamic of burden and professional growth [38].

Regarding the therapeutic alliance, some participants perceived no difference in bond quality between online and in-person sessions, while others felt that the digital interface might limit the depth of the connection. This ambivalence is mirrored in the literature, with some studies indicating possible barriers to building alliances online [42,43], and others suggesting that online psychotherapy can be equally effective [40,44]. A qualitative study with Italian psychologists and psychotherapists during the COVID-19 pandemic [45] found that online interventions deeply affected perceptions of the therapeutic relationship. While many professionals described a sense of continuity and even unexpected closeness in some cases, others reported feelings of detachment and difficulties in emotional attunement due to the absence of physical presence. The transition to remote therapy challenged therapists to renegotiate boundaries, redefine presence, and actively construct a new sense of intimacy through the screen. These findings emphasize that online settings do not inherently weaken therapeutic bonds but require deliberate relational work to preserve and adapt them [45].

Participants reported a lack of specific training to deal with suicidal behavior, which led to a sense of helplessness and emotional distress. The literature corroborates this challenge, highlighting that insufficient training on suicide prevention remains a significant gap in mental health education, which impacts both professional and personal domains [30,46].

Participants’ perceptions of the incidence of suicidal behavior during the COVID-19 pandemic varied, with some believing there had been an increase, while others believed there had been a decrease. Although numerous studies indicate an increase in rates of suicidal behavior during the pandemic [10,11,12,13,14,15], different regions, contexts, and population groups showed variations [8], which may account for divergent perceptions or experiences among the clinical psychologists interviewed.

In terms of clinical practice, the importance of a solid therapeutic bond was emphasized by the professionals, regardless of the care modality. The literature reinforces that comprehensive suicide risk assessments must consider both predisposing and precipitating factors in order to identify the need for intensive monitoring and interventions [7].

When discussing online psychotherapy during the pandemic, participants reported concerns about the effectiveness of the online format for suicidal patients, including difficulties related to internet instability, privacy, and loss of non-verbal communication. These are consistent with broader concerns raised in studies on online mental health care [19,42,44,47,48]. Karekla et al. [42] explain that a lack of technological proficiency may lead patients to disengage from online care.

The emotional toll reported by professionals in the present study—marked by feelings of discomfort, anguish, worry, tension, insecurity, overload, and even despair—reflects the intense psychological demands involved in managing suicidal behavior in online psychotherapy. While some of these reactions were attributed to the limitations of the virtual format, such as physical distance and reduced access to nonverbal cues, it is important to note that these emotional responses are not uncommon among mental health professionals working with suicidal patients in face-to-face settings as well [49].

This suggests that the distress experienced may stem not only from the medium of care, but also from the inherent complexity and emotional weight of working with high-risk clinical scenarios. These findings point to the importance of structured support systems, supervision, and training—regardless of the care modality—to help professionals navigate the emotional demands of suicide risk management effectively [19]. The literature emphasizes the role of self-care and support networks in maintaining therapist wellbeing and treatment quality in high-stress contexts [49].

Professionals who lost patients to suicide described experiences of grief and professional suffering. This finding aligns with studies that underscore the importance of postvention strategies such as psychotherapy and peer support [17]. In spite of this, positive feelings such as hope and relief arising from the realization that it is possible to help patients even in an online context were also described. Literature supports the notion that working with individuals who exhibit suicidal behavior can lead to a sense of satisfaction, particularly when professionals perceive positive outcomes [49].

Some professionals reported that local health services were inaccessible or insufficient for suicide risk situations, requiring them to assume greater responsibility. These findings point to structural deficiencies in mental health networks, particularly in under-resourced settings [42].

Despite these difficulties, some professionals highlighted strategies that were successfully implemented in online settings, such as increased flexibility in scheduling, availability for support messages, and building a therapeutic bond that emphasized accessibility. These actions are supported by the literature, indicating the importance of adapting traditional therapeutic approaches to ICT environments [42]. However, these strategies—though effective—were improvised, highlighting the urgent need for formal training, supervision, and systematized protocols to support clinicians managing suicide risk in digital contexts. Such resources are essential to reduce the emotional burden on professionals and ensure ethical, safe, and sustainable care.

One participant in the study compared online interventions for patients with suicidal behavior to the emotional support for crisis situations offered by CVV. Another Brazilian researcher made the same comparison, based on his CVV experience, which took place 25 years prior to the pandemic, noting that telephone support was effective and relevant, with a high likelihood of preventing suicides through adequate support during critical moments. Thus, he concludes that telephone support manifests undeniable psychotherapeutic effects [47].

Ethical considerations, including breaches of confidentiality to ensure patient safety, were described. Mapping the patient’s location, assessing access to lethal means, and involving support networks were strategies aligned with clinical guidelines for ICT-mediated crisis care [42].

Other advantages of online psychotherapy were also acknowledged. Participants emphasized the increased accessibility of care, especially for individuals in emotional distress, remote areas, or socially stigmatized contexts. Moreover, some psychologists described the continuity of care as an important benefit of online therapy, particularly for patients with depressive symptoms who might otherwise disengage. These aspects are consistent with research that points to digital health interventions as promising tools for expanding mental health access and reducing suicide risk [30,42,50]. The flexibility provided by online formats supports the literature indicating that digital interventions can help overcome access barriers, particularly in low- and middle-income countries, which suffer from a shortage of adequate treatment options [7,19,42,47,50].

Furthermore, the use of online resources even when the patient is seen in person remains a possibility and a tool, which aligns with the Brazilian Psychiatric Association’s directives, which stress the importance of rapid therapeutic intervention for patients in suicidal crisis, ideally within minutes to a few hours [7,17]. Despite the recommendation that psychologists maintain some level of availability during imminent suicide situations, studies recommend establishing clear guidelines for interactions in online contexts, emphasizing that professionals should not be perpetually available and should limit out-of-session contacts to avoid role confusion [47].

Despite the optimistic outlook of the participants regarding online psychotherapy for patients with suicidal behavior, one study found no evidence that ICT-mediated cognitive-behavioral therapy reduced suicidal ideation in adults, suggesting that further research is needed to identify effective models, especially in high-risk case [17]. As there were no participants from this theoretical approach in the present study, it was not possible to investigate whether the perceptions aligned with the results of the aforementioned research.

Finally, participants expressed their intention to continue offering online psychotherapy beyond the pandemic, recognizing it as a viable format for suicide prevention. This aligns with projections that digital mental health care will become increasingly common in the coming years: researchers estimate that, within the next decade, individual psychotherapy sessions delivered via videoconferencing could constitute between 55% and 70% of total consultations [51]. However, some clinical psychologists who participated in this study still prefer in-person formats for addressing suicidal behavior, suggesting that flexibility and clinical judgment are essential in choosing the appropriate modality [19].

## 5. Final Considerations

The study’s limitations include the absence of participants from theoretical frameworks other than psychoanalysis and phenomenology and the exclusive focus on Brazilian psychologists from the state of São Paulo, which may not reflect the breadth of experiences in other regions.

The results of this research prompt intriguing questions: Did the provision of online psychotherapy for patients with suicidal behavior contribute to curbing the rise in suicide rates during and after the pandemic? What are the perceptions of patients with suicidal behavior regarding the significance and effectiveness of online psychotherapy? Further research on these topics is recommended. In addition, statistical and quantitative investigations could be conducted to complement psychologists’ insights and validate the applicability of the theoretical model in different contexts.

The practical implications of this research highlight the necessity for adequate training for professionals and for psychology students, equipping them to manage patients exhibiting suicidal behavior and providing supervised experiences with online psychotherapy, as the transformations initiated during the pandemic will continue to evolve, requiring them to be prepared. It is hoped that the successful strategies developed and described by the clinical psychologists interviewed in this study can contribute to the construction of references for professional practice.

## 6. Conclusions

Drawing from 20 interviews conducted with 10 Brazilian psychologists, a coherent dataset was constructed and analyzed in two stages to illuminate clinical practices involving patients with suicidal behavior during the pandemic. In conclusion, the findings suggest that online psychotherapy in such cases is a complex and context-sensitive process that requires thoughtful clinical judgment. Many participants perceived it as a viable—and at times, promising—resource, capable of offering meaningful possibilities when conditions are favorable for both patients and clinical psychologists.

## Figures and Tables

**Table 1:** Outline of the theoretical model, categories, and subcategories.

Theoretical Model Pathways of Care: Main Roads and Access Routes in Online Psychotherapy for Suicidal Behavior	
Categories	Subcategories
Section 3.1. Reflecting on Suicidal Behavior	Section 3.1.1. General Experiences in Managing Suicidal Behavior Section 3.1.2. Suicidal Behavior During the Pandemic
Section 3.2. Addressing Specificities of Online Interventions	Section 3.2.1. Challenges, Difficulties, and Insecurities Section 3.2.2. Psychologists’ Feelings on the Online Management of Suicidal Behavior
Section 3.3. Managing Suicidal Behavior in Online Psychotherapy	Section Strategies and Resources Used
Section 3.4. Evaluating Online Psychotherapy for Patients with Suicidal Behavior	Section 3.4.1. Perceived Facilities and Advantages Section 3.4.2. Disadvantages Identified Section 3.4.3. General Assessment

## Data Availability

The data supporting the findings of this study are available from the corresponding author upon reasonable request for the purpose of peer review. However, the data cannot be made publicly available due to ethical considerations and the need to protect the confidentiality and privacy of the study participants.

## Abbreviations

The following abbreviations are used in this manuscript:

CFP	Federal Council of Psychology
GT	Grounded Theory
CRP	Regional Psychology Councils
ICF	Informed Consent Form
CAAE	Certificate of Presentation for Ethical Consideration
COREQ	Consolidated Criteria for Reporting Qualitative Research
ICT	Information and Communication Technology
CVV	Centro de Valorização da Vida
CAPES	Coordenação de Aperfeiçoamento de Pessoal de Nível Superior
PROEX	Programa de Excelência Acadêmica
CNPq	Conselho Nacional de Desenvolvimento Científico e Tecnológico
